# Modulating the p53-MDM2 pathway: the therapeutic potential of natural compounds in cancer treatment

**DOI:** 10.17179/excli2024-7791

**Published:** 2024-11-22

**Authors:** Iman Ramli, Thamere Cheriet, Anna Maria Posadino, Roberta Giordo, Grazia Fenu, Kingsley Chukwuemeka Nwachukwu, Oluwafemi Adebayo Oyewole, Charles Oluwaseun Adetunji, Daniela Calina, Javad Sharifi-Rad, Gianfranco Pintus

**Affiliations:** 1 Laboratory of Applied Biochemistry, Faculté des Sciences de la Nature et de la Vie, Université Constantine 1 Frères Mentouri, 25000 Constantine, Algeria; 2 Unité de Valorisation des Ressources Naturelles, Molécules Bioactives et Analyses Physicochimiques et Biologiques, Université Constantine 1 Frères Mentouri, 25000 Constantine, Algeria; 3 Département Science de la Nature et de la Vie, Faculté des Sciences Exactes et Sciencede la Nature et de la Vie, Université Mohammed Boudiaf-Oum El-Bouaghi, 04000 Oum El-Bouaghi, Algeria; 4 Department of Biomedical Sciences, University of Sassari, Viale San Pietro 43B, 07100 Sassari, Italy; 5 Department of Microbiology, Faculty of Biological Sciences, Abia State University, Uturu, Nigeria; 6 Covenant Applied Informatics Communication African Centre for Excellence (CApIC-ACE), Covenant University, Ota, Ogun State, Nigeria; 7 Department of Microbiology, Federal University of Technology, Minna, Nigeria; 8 Laboratory of Applied Microbiology, Biotechnology and Nanotechnology, Department of Microbiology, Edo State University, Uzairue, Edo State, Nigeria; 9 Department of Clinical Pharmacy, University of Medicine and Pharmacy of Craiova, 200349 Craiova, Romania; 10 Universidad Espíritu Santo, Samborondón 092301, Ecuador; 11 Centro de Estudios Tecnológicos y Universitarios del Golfo, Veracruz, Mexico; 12 Department of Medicine, College of Medicine, Korea University, Seoul 02841, Republic of Korea; 13 Department of Medical Laboratory Sciences, College of Health Sciences and Sharjah Institute for Medical Research, University of Sharjah, 27272 Sharjah, United Arab Emirates

**Keywords:** p53, MDM2, cancer therapy, natural products, tumor suppression

## Abstract

The p53-MDM2 pathway plays a crucial role regulating tumor suppression and is a focal point of cancer research. This literature review delves into the complex interplay between the tumor suppressor protein p53 and its main regulator MDM2, highlighting their interaction and implications in cancer development and progression. The review compiles and summarizes the existing understanding of the biology and regulation of p53 and MDM2, emphasizing their roles in various cellular processes, including cell cycle regulation, DNA repair, apoptosis, and metabolism. It also discusses the disruption of the p53-MDM2 interaction in different cancer types and its impact on tumor progression and therapeutic resistance. Recent studies have highlighted natural products as promising avenues for targeting the p53-MDM2 pathway. This review categorizes these natural compounds into three groups based on their mechanisms: those that inhibit MDM2 expression or stability, those that prevent p53-MDM2 binding, and those that stabilize p53 by inhibiting MDM2's E3 ligase activity. Detailed mechanisms of action, structural aspects, and potential therapeutic applications of various natural products, including polyphenols, terpenoids, and alkaloids, are presented. Despite significant advances in understanding the p53-MDM2 interaction and the identification of natural products capable of modulating this pathway, further research is necessary to evaluate the clinical efficacy, toxicity, and bioavailability of these compounds. The promising role of natural products in cancer therapy underscores the importance of ongoing investigation and development of these agents as potential treatments for cancer.

## Abbreviation list

Ac: Acetylation

AKT: Ak mouse strain thymoma protein

ARF: ADP ribosylation factor

Bax: Bcl-2-associated X protein

Bcl-xL: B-cell lymphoma-extra large

Bcl-2: B-cell lymphoma 2 protein

c-FLIP: Cellular FLICE-like inhibitory protein

CDK: Cyclin-dependent kinase

CK2: Casein kinase 2

CSCs: Cancer stem cells

CTD: C-terminal domain

DNA-PK: DNA-dependent protein kinase

EGFR: Epidermal growth factor receptor

EGF: Epidermal growth factor

ER: Endoplasmic reticulum

E2F: E2F transcription factor

ETS2: E26 transformation-specific transcription factor 2

FOXO3a: Forkhead box O3

GADD45: Growth arrest and DNA-damage-inducible protein 45

HCC: Hepatocellular carcinoma

HDM2: Human double minute 2 homolog (MDM2)

HSP: Heat shock protein

HSP90: Heat shock protein 90

JAK1/2: Janus kinase 1/2

MAPK: Mitogen-activated protein kinase

MCL: Myeloid cell leukemia

MDM2: Murine double minute 2

mTOR: Mechanistic target of rapamycin

NADH: Nicotinamide adenine dinucleotide (reduced)

NFAT1: Nuclear factor of activated T cells 1

NF-κB: Nuclear factor kappa-light-chain-enhancer of activated B cells

NLS: Nuclear localization signal

NoLS: Nucleolar localization signal

NQO1: NAD(P)H quinone oxidoreductase 1

PARP: Poly (ADP-ribose) polymerase

PI3K: Phosphoinositide 3-kinase

p21: Cyclin-dependent kinase inhibitor 1

p27: Cyclin-dependent kinase inhibitor 1B

p53: Tumor suppressor 53

PML: Promyelocytic leukemia protein

PTEN: Phosphatase and tensin homolog

pRb1: Retinoblastoma protein 1

RING: Really interesting new gene

ROS: Reactive oxygen species

SIRT1: Sirtuin 1

SNP: Single nucleotide polymorphism

TGF-β: Transforming growth factor beta

TP53: Tumor protein p53

TRAIL: TNF-related apoptosis-inducing ligand

VEGF: Vascular endothelial growth factor

VGFA: Vascular endothelial growth factor A

XIAP: X-linked inhibitor of apoptosis protein

## Introduction

During a malignant transformation, the accumulation of genomic lesions leads to alterations in gene expression, cell signaling, and cell cycle progression. These phenomena generally define the malignant nature of a cell, indicating sustained proliferative capacity, evasion of growth suppressors, resistance to cell death and replicative senescence, enhanced angiogenesis, and the initiation of invasion and metastasis (Iqbal et al., 2024[91]). A major focus in cancer research is the activation of oncogenes and the inactivation of tumor suppressor genes, both of which play important roles in cancer initiation and progression (Morla-Barcelo et al., 2024[144]). These processes are also recognized as potential targets for cancer therapy. Among tumor suppressors, p53 has been the most extensively studied over recent decades. Known as the “guardian of the genome”, p53 is a potent transcription factor that responds to oncogenic stresses and environmental insults by inducing a series of mechanisms, including senescence, apoptosis activation, and cell cycle arrest. These mechanisms ensure that damaged cells are either repaired or eliminated before they undergo irreversible transformation into cancer cells (K et al., 2024[96]; Liu et al., 2024[125]; Rusin, 2024[190]). The murine double minute 2 (MDM2) is a ubiquitin ligase that keeps low levels of p53 in normal cells (García-Cano et al., 2020[59]). MDM2 serves as the principal p53 regulator in a negative-feedback loop, where p53 initiates MDM2 expression, which in turn induces the monoubiquitinated degradation of p53, leading to the quenching of p53 biological activity (García-Cano et al., 2020[59]; Rusin, 2024[190]). In humans, the TP53 gene encodes p53, while Trp53 encodes it in mice. Mutations in p53 are found in approximately 50 % of human cancers, while amplification of the MDM2 gene occurs in around 17 % of tumors, either in the presence or absence of p53 mutations. Both conditions are associated with poor prognosis and resistance to chemotherapy (Momand et al., 1998[143]; Zhang and Wang, 2000[265]; Levine and Oren, 2009[115]). Consequently, targeting the MDM2-p53 linkage represents an encouraging strategy for cancer therapy. This review offers a novel and detailed exploration of natural compounds as modulators of the p53-MDM2 pathway, an important target in cancer therapy. This comprehensive review categorizes these compounds based on their mechanisms - such as inhibiting MDM2 expression, preventing p53-MDM2 binding, and stabilizing p53. By focusing on the therapeutic potential of natural agents, it presents a unique perspective on developing more effective and less toxic cancer treatments.

## Review Methodology

An extensive literature investigation was conducted on different databases, including Scopus, PubMed/MedLine, TRIP databases. The search terms included a combination of keywords related to cancer and natural compounds, focusing on the p53-MDM2 interaction. The specific search strings used were: (tumor OR tumors OR cancer OR cancers OR neoplasms OR neoplasm OR proliferation OR antiproliferative OR metastasis OR metastatic OR angiogenesis OR carcinoma OR growth OR malignancy OR tumor suppressor OR oncogene OR division OR oncoprotein OR prevention OR *in vitro* OR *in vivo* OR treatment); (natural compounds OR natural antioxidants OR polyphenols OR terpenoids OR alkaloids); (p53 OR MDM2 OR p53-MDM2 interaction). To ensure the relevance and quality of the studies included in this review, we implemented the following inclusion and exclusion criteria:

### Inclusion criteria:

- Studies published in peer-reviewed journals
- Articles written in English
- Research focused on the p53-MDM2 interaction in cancer
- Studies investigating natural compounds (e.g, polyphenols, terpenoids, alkaloids) with potential anticancer properties
- Studies providing mechanistic insights, including molecular and cellular effects of the natural compounds
- Both *in vitro *and* in vivo* studies.

### Exclusion criteria:

- Non-peer-reviewed articles, including reviews, editorials, and opinion pieces
- Studies not focused on the p53-MDM2 interaction
- Research involving synthetic compounds or non-natural products
- Articles not available in English
- Studies lacking detailed methodological descriptions.

To ensure the accuracy and reliability of the data, the taxonomy of plant species mentioned in the included studies was validated using the World Flora Online (WFO) database. Additionally, the chemical structures of the natural compounds were verified using PubChem. The most representative data from the included studies have been synthesized and presented in tables and figures to facilitate a clear understanding of the findings. These visual aids summarize key information on mechanistic insight, structural aspects, and potential therapeutic approaches of various natural products targeting the p53-MDM2 pathway.

## p53-MDM2 Biology, Regulation and Interaction: Connecting the Dots

Extensive research on p53 and its regulatory network has revealed a high level of complexity. Beyond its well-established role in cancer, p53 is also implicated in the pathogenesis of several diseases, including cardiovascular and infectious diseases, neurodegenerative and metabolic disorders, and autoimmune conditions (Takatori et al., 2014[215]; Siegl and Rudel, 2015[206]; Kung and Murphy, 2016[106]; Szybińska and Leśniak, 2017[214]; Aloni-Grinstein et al., 2018[2]; Maor-Nof et al., 2021[132]; Men et al., 2021[135]). Additionally, P53 was also found to be essential in driving the pathologic effects of COVID-19 (Cardozo and Hainaut, 2021[25]). The expanding understanding of p53's involvement in these diverse pathologies has provided deeper insights into the molecular mechanisms influenced by this protein, including its roles in metabolism, autophagy, translational regulation, and epigenetic control (Levine, 2019[114]; Boutelle and Attardi, 2021[18]).

### p53 biology: the guardian of the genome

**Figure 1:**
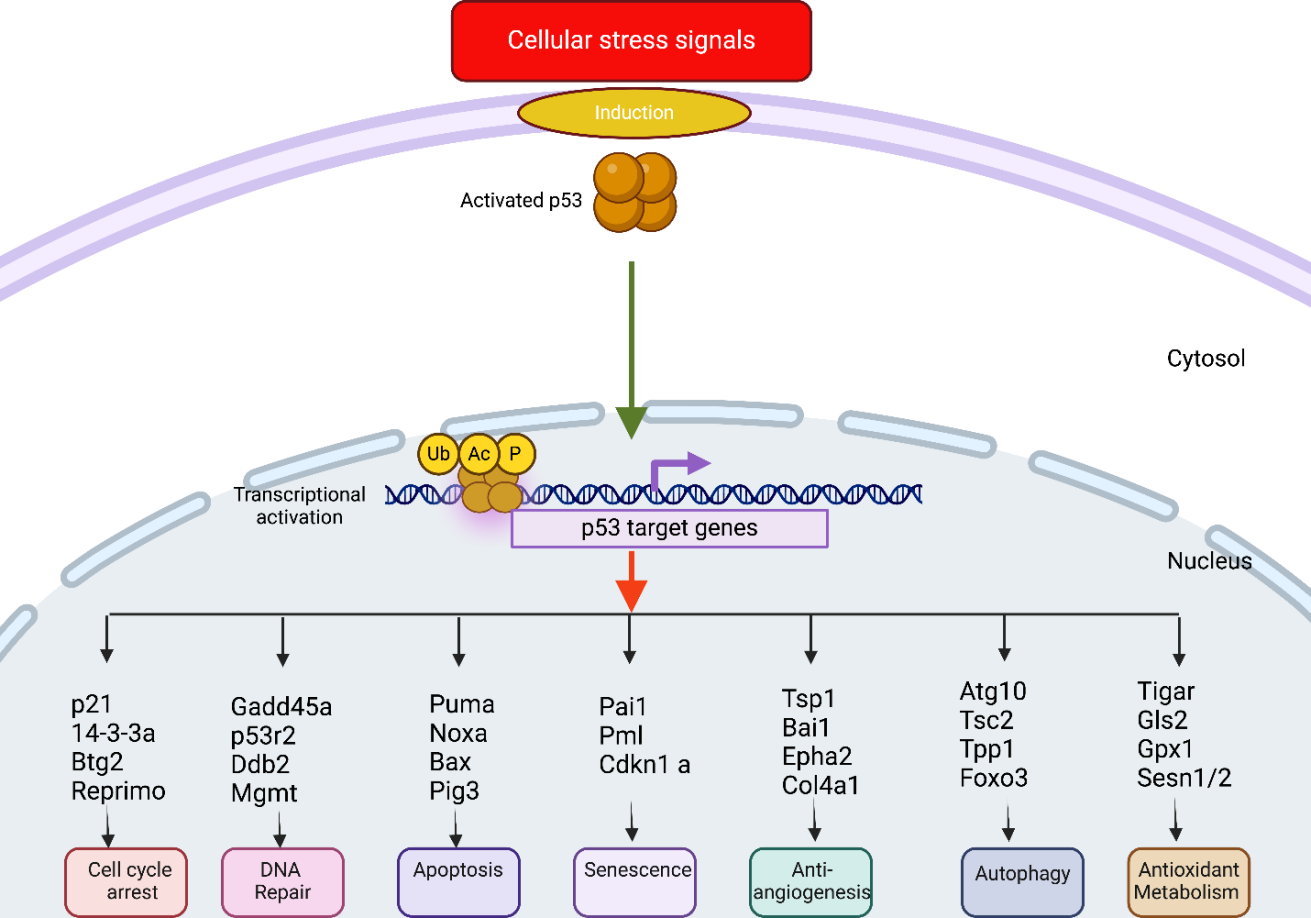
p53 functions against cellular stress signals and target genes. In response to various inducers of cellular stress, the activation of p53 results in its post-translational via phosphorylation (P), acetylation (Ac), or ubiquitination (Ub). This activation contributes to the induction of multiple genes implicated in different cellular processes via regulation of downstream targets and/or signaling pathways, including apoptosis, cell cycle arrest, DNA repair…etc. p21: cyclin-dependent kinase inhibitor 1, 14-3-3a: 14-3-3a protein, Btg2: B-cell translocation gene 2, Reprimo: Reprimo gene, Gadd45a: Growth arrest and DNA-damage-inducible gene, p53r2: p53 inducible ribonucleotide reductase gene, Ddb2: Damage Specific DNA Binding Protein 2 coding gene, Mgmt: Methylguanine methyltransferase coding gene, Puma: P53 Upregulated Modulator of Apoptosis coding gene, Noxa: axotomy-induced motor neuron death, Bax: Bcl-2-associated X coding gene, Pig3: p53 inducible gene 3, Pai1: plasminogen activator inhibitor 1, Pml: Promyelocytic leukemia protein gene, Cddkn1 a: Cyclin Dependent Kinase Inhibitor 1a, Tsp: thrombospondine 1, Bai 1: Brain-specific angiogenesis inhibitor 1, Epha2: ephrin type-A receptor 2 coding gene, Col4a1: Collagen type IV alpha 1 Chain, Atg 10: ATG10 autophagy related 10, Tsc 2: tuberous sclerosis complex 2 gene, Tpp1: Tripeptidyl-peptidase 1 coding gene, Foxo3: transcription factor forkhead box O-3, Tigar: TP53 Induced Glycolysis regulatory phosphatase, Gls2 Glutaminase 2, Gpx1: Glutathione peroxidase 1, Sesn1/2: Sestrin 1 and 2 coding gene

It is well-known that the regulatory network governing p53 functions is complex and influenced by a multitude of molecular factors. These include the mutation status and post-translational modifications of p53, the response elements (REs) of p53-target genes, the interactions between p53 and its cofactors, as well as the dynamic heterogeneity of p53 activity (Hafner et al., 2017[75]; Farkas et al., 2021[50]). The process of controlling cell fate is remarkably orchestrated by a vast array of p53-target genes and mechanisms, numbering over 3,500 (Figure 1(Fig. 1)) (Fischer, 2017[52]; Sammons et al., 2020[192]). Primarily, p53 acts as a tumor suppressor by preventing malignant transformation in cells. It achieves this by activating the transcription of targeted genes, which then produce proteins that trigger apoptosis, cell growth arrest, or senescence in response to stress signals (Vousden and Prives, 2009[234]). Additionally, p53 plays a key role in regulating DNA repair systems and is responsible for upregulating genes involved in cell cycle progression, including those related to cell cycle checkpoints and genomic integrity. This regulation facilitates the initiation of cell cycle arrest and/or apoptosis in the presence of DNA damage (Menendez et al., 2009[136]).

In case of genotoxicity and/or genomic instability due to ionizing radiations or chemotherapy, induced p53, elevated levels of p53 work to repair cellular damage by increasing the expression of pro-apoptotic (BAX and PUMA) and cell cycle proteins (GADD45, p21) (Steffens Reinhardt et al., 2023[209]). The induction of p21 and/or GADD45 inhibits the activity of CDC2/cyclin E, halting mitosis and leading to cycle arrest in the G_2_/M phase (Shangary and Wang, 2009[198]). Another way p53 suppresses tumorigenesis is through the p21-Rb-E2F pathway, which triggers cell senescence in response to oxidative stress, DNA damage, or telomere erosion (Steffens Reinhardt et al., 2023[209]). p53 also promotes base excision repair by upregulating components such as Ape/ref1, OGG1, and Polβ. Additionally, p53 enhances the expression of Ku70, which interacts with BAX, promoting its translocation to the mitochondria, oligomerization, and subsequent cell survival. Other DNA repair systems upregulated by p53 are the mismatch repair and nucleotide excision repair components (Menendez et al., 2009[136]). Beyond these roles, p53 acts as a transcriptional repressor for several genes, including c-fos, myc, VEGF-A, and genes associated with cell survival, all of which are involved in promoting pathways related to survival, proliferation, and angiogenesis (Ginsberg et al., 1991[63]; Zhang et al., 2000[264]; Menendez et al., 2009[136]). Furthermore, p53 regulates the transcription of microRNAs, particularly members of the miR-34 family. Increased levels of miR-34a induced by p53 enhance apoptosis and influence the expression of genes related to DNA repair, apoptosis, cell cycle regulation, and angiogenesis (Fu et al., 2023[55]). These findings highlight p53 as a key regulator that links various cellular and molecular signaling pathways, playing a crucial role in processes such as apoptosis, senescence, cell cycle control, angiogenesis, metabolism, immune response, cell motility, differentiation, migration, and cell-cell communication (Amendolare et al., 2022[5]; Wang et al., 2023[235]).

### MDM2 biology: the loyal companion of p53

**Figure 2:**
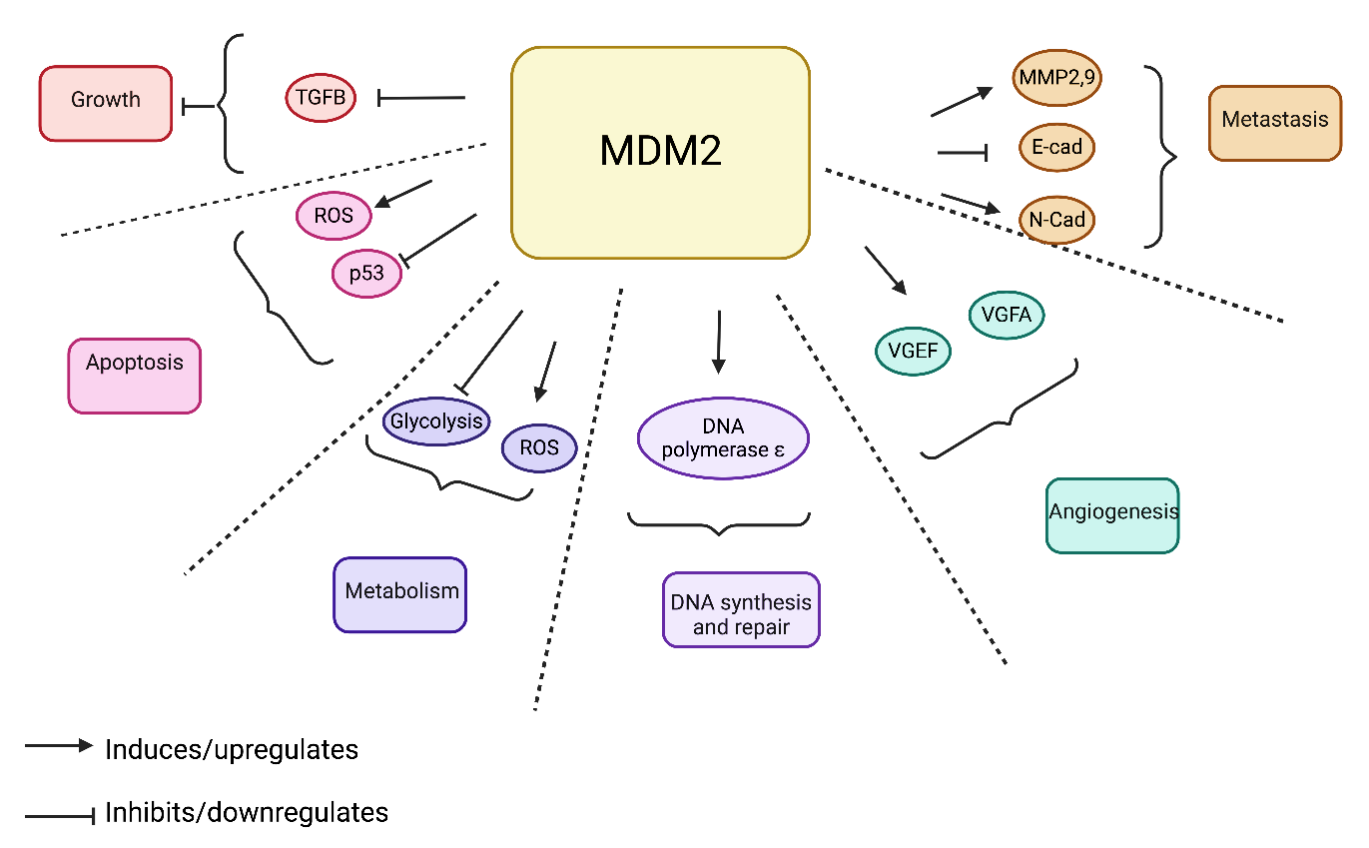
MDM2 functions and targeted cellular processes. The diagram illustrates the diverse roles of MDM2 in cancer progression and cellular processes. MDM2, a fundamental regulator of the p53 tumor suppressor, influences multiple cellular pathways. It inhibits growth via downregulating TGF-β and affects apoptosis through reactive oxygen species (ROS) and p53 modulation. MDM2's role in metabolism includes the regulation of glycolysis and ROS levels. In DNA synthesis and repair, MDM2 interacts with DNA polymerase ε. MDM2 also promotes metastasis by upregulating MMP2/9 and E-cadherin (E-cad) and downregulating N-cadherin (N-cad). Additionally, it contributes to angiogenesis by regulating VGFA and VGEF. The arrows indicate whether MDM2 induces/upregulates (black arrows) or inhibits/downregulates (black lines) specific processes and molecules. The dashed lines represent the interconnected pathways influenced by MDM2, emphasizing its central role in cancer biology.

Elevated levels of MDM2 have been correlated with poor prognosis in several cancer types, including solid tumors of the lung, esophagus, breast, stomach, as well as liposarcomas, glioblastomas, and leukemias (Yao et al., 2024[256]). Various molecular mechanisms contribute to MDM2 overexpression in these cancers, most notably mdm2 gene amplification (Momand et al., 1998[143]). Additionally, a single nucleotide polymorphism at position 309 (SNP309) in the MDM2 gene promoter has been shown to enhance transcription and translation (Yao et al., 2024[256]). MDM2 overexpression is also associated with metastasis and advanced stages of cancers such as osteosarcoma, colon, breast, and prostate cancers, and has been linked to chemotherapy resistance (Lin et al., 2024[123]). During tumorigenesis, MDM2 plays a multifaceted role, regulating key cellular processes such as the cell cycle, apoptosis, angiogenesis, metastasis, metabolism, and DNA synthesis and repair (Zafar et al., 2023[258]) (Figure 2(Fig. 2)).

The MDM2 oncoprotein is regulated through several well-characterized mechanisms (Rayburn et al., 2009[184]). A key regulatory pathway involves p53-induced transcription of mdm2 via the P2 promoter, while the P1 promoter drives basal transcription independently of p53 (Barak et al., 1994[12]). Other transcription factors, including NFAT1 (Zhang et al., 2012[266]), NF-κB (Thomasova et al., 2012[221]), IRF-8, SP1 (Rayburn et al., 2009[184]), Fli-ETS (Truong et al., 2005[223]), and Ras/Raf/MEK/MAPK (Ries et al., 2000[186]), positively modulate MDM2 expression through both the P1 and P2 promoters. Conversely, the tumor suppressor PTEN acts as a negative regulator of MDM2, independent of p53 (Ries et al., 2000[186]). Additionally, microRNAs (miRNAs) such as miR-29, miR-18b, miR-145, and miR-143 initiate epigenetic mechanisms that downregulate MDM2 by inhibiting its mRNA translation (Dar et al., 2013[37]; Zhang et al., 2013[262]). Post-translational modifications, such as phosphorylation, also play an important role in MDM2 regulation. For instance, modifications by the ATM protein reduce MDM2 stability (de Toledo et al., 2000[38]; Maya et al., 2001[133]; Meulmeester et al., 2005[139]), while the Akt pathway facilitates MDM2 translocation from the cytoplasm to the nucleus, promoting p53 degradation (Mayo and Donner, 2001[134]; Zhou et al., 2001[274]; Ogawara et al., 2002[155]; Gama et al., 2009[57]). Other enzymes, such as CK2, DNA-PK, and components of the Ras/Raf/MEK/ MAPK pathway, further regulate MDM2 function (Rayburn et al., 2009[184]). Extensive evidence underscores that MDM2 is a key regulator of multiple cellular processes, independent of its interaction with p53. MDM2 influences DNA synthesis and repair through interactions with DNA polymerase ε (Vlatkovic et al., 2000[231]; Asahara et al., 2003[7]), DHFR (Maguire et al., 2008[129]), centrosome amplification (Carroll et al., 1999[26]), and the MRN DNA repair complex, including Nbs1 (Alt et al., 2005[4]; Bouska et al., 2008[17]). Additionally, MDM2 interacts with cellular proteins such as DNMT3A (Tang et al., 2012[218]), the Rb/E2F-1 complex (Hsieh et al., 1999[82]; Katsube et al., 2003[100]; Uchida et al., 2005[225]), MTBP (Boyd et al., 2000[19]; Brady et al., 2005[20]), p107 (Dubs-Poterszman et al., 1995[43]), and the cyclin-dependent kinase inhibitor p21, promoting cell cycle progression, particularly through the S-phase (Zhang et al., 2004[269]; Xu et al., 2010[249]). MDM2 also plays a role in apoptosis inhibition by modulating both pro-apoptotic and anti-apoptotic proteins. It interacts with the E2F1/Rb pathway (Bouska et al., 2008[17]) and apoptosis mediators such as p73 (through p73 NEDDylation, which prevents p53 transactivation) (Bouska et al., 2008[17]; Malaguarnera et al., 2008[130]), and FOXO3a (by reducing its stability) (Fu et al., 2009[56]). MDM2 further enhances anti-apoptotic signaling by upregulating XIAP, which inactivates caspase-mediated apoptosis (Gu et al., 2009[73]). Beyond its function as a negative regulator of p53, MDM2 is involved in the regulation of proteins important for DNA repair, apoptosis, cell dynamics, and invasion pathways (Bouska et al., 2008[17]; Rayburn et al., 2009[184]; Manfredi, 2010[131]; Li and Lozano, 2013[120]). While the intricate web of MDM2 interactions, both dependent and independent of p53, highlights its multifaceted role in cellular regulation, the MDM2-p53 axis remains central to maintaining normal cellular homeostasis. Over the past decades, research has primarily focused on understanding the MDM2 interactions that influence the cellular and molecular levels of p53, both directly and indirectly (Subhasree et al., 2013[212]).

### p53-MDM2 interaction: a tremendous complexity and complementarity

**Figure 3:**
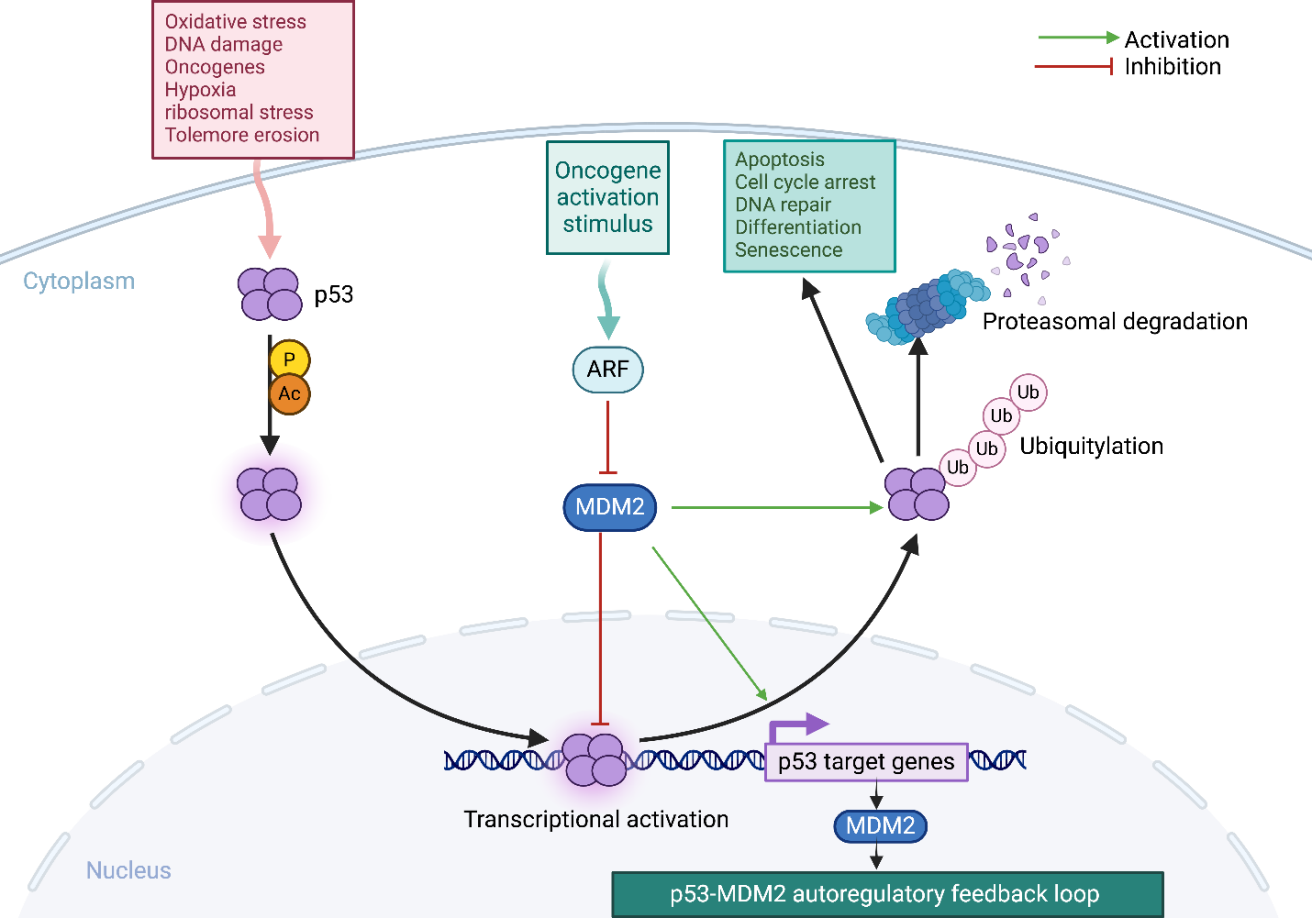
p53-MDM2 interaction. p53 and MDM2 are part of an autoregulatory feedback loop. p53 increases MDM2 expression, which in turn inhibits p53 activity by promoting its degradation in the nucleus and cytoplasm, inhibiting its transcriptional activity, and facilitating its nuclear export. Various DNA-damaging agents or oncogene inhibitors trigger p53 activity. DNA damage leads to p53 phosphorylation and enhances MDM2, preventing p53 interaction and ubiquitination. In parallel, activated oncogenes induce ARF protein, leading to the sequestration of MDM2 into the nucleus, hence blocking p53 degradation. In contrast, survival signals regulate the nuclear transport of MDM2 via the Akt pathway, leading to the destabilization of p53. p53: Tumor suppressor 53, MDM2: The murine double minute 2, P: phosphorylation, Ub: Ubiquitination, Ac: Acetylation, Arf: ADP ribosylation factor

The relationship between MDM2 and p53 is primarily built upon the regulation of the latter and serves as a crucial checkpoint for most stress-mediated signaling pathways that lead to p53 activation and regulation (Levine, 2020[113]). It is well-established that p53 plays a central role in preventing the proliferation of abnormal cells with genetic instabilities. Under normal conditions, p53 levels are kept low by MDM2 to maintain cellular homeostasis (Moll and Petrenko, 2003[142]). In this section, we explore the unique MDM2-p53 interaction, which has been extensively studied as a pivotal process in cancer therapeutics. MDM2 tightly controls a rapid regulatory mechanism that toggles the activation of wild-type (WT) p53 on and off. One of the primary mechanisms by which transformed cells drive tumorigenesis is through the overexpression of MDM2, which inhibits p53's transcriptional activity and reduces its cellular levels (Cahilly-Snyder et al., 1987[22]; Fakharzadeh et al., 1991[46]; Oliner et al., 1993[157]). Reduced MDM2 activity results in p53 mono-ubiquitination and its export from the nucleus, while increased MDM2 activity leads to poly-ubiquitination and subsequent degradation of p53 in the nucleus (Fang et al., 2000[49]; Rodriguez et al., 2000[188]; Lai et al., 200[109]; Lee and Gu, 2010[111]). A widely supported hypothesis regarding the negative autoregulatory loop between p53 and MDM2 is the amplification of the MDM2 gene observed in several human sarcomas with wild-type p53 (Momand et al., 1998[143]). At the molecular level, MDM2 binds to p53 via a primary N-terminal p53-binding domain and contains a C-terminal RING domain, which functions as an E3 ubiquitin ligase. This domain, along with various sequence motifs such as the nucleolar localization signal (NoLS), nuclear localization signal (NLS), and nuclear export signal (NES), facilitates MDM2's localization within the nucleus and its export (Chi et al., 2005[34]; Yu et al., 2006[257]; Poyurovsky et al., 2011[170]). The interaction between MDM2 and p53 is further strengthened by the ability of p53 to bind MDM2 at multiple sites. Through its C-terminal RING domain, MDM2 ubiquitinates p53, leading to its degradation via the proteasome pathway (Haupt et al., 1997[76]; Midgley et al., 2000[140]). However, stress-mediated signaling pathways can disrupt the MDM2-p53 interaction through various mechanisms. These pathways often involve post-translational modifications (PTMs) of p53, such as phosphorylation at serine residues 15, 20, 37, and 106, and threonine 18, which weaken the MDM2-p53 interaction. Acetylation at lysine residues in the C-terminal domain (CTD) of p53 further prevents MDM2-mediated ubiquitination (Shieh et al., 1997[203]; Unger et al., 1999[227]; Nakamura et al., 2000[150]; Rodriguez et al., 2000[188]; Sakaguchi et al., 2000[191]; Li et al., 2002[117]; Hsueh et al., 2013[85]). Additionally, MDM2 can export p53 out of the nucleus (Haupt et al., 1997[76]; Honda et al., 1997[78]), preventing its interaction with transcriptional co-activators (Oliner et al., 1993[157]) and enhancing the transcription of p53 co-repressors (Wu et al., 1993[247]; Thut et al., 1997[222]; Chi et al., 2005[34]). In turn, wild-type (WT) p53 promotes the transcription of MDM2, establishing a regulatory feedback loop (Barak et al., 1994[12]). This loop finely tunes p53 functions through p53-mediated regulation of MDM2 at its promoter (Barak et al., 1994[12]; Shangary and Wang, 2009[198]; Vousden and Prives, 2009[234]). The complexity of this autoregulatory loop ensures the maintenance of physiological p53 levels in normal cells, as the precise homeostatic concentration of p53 is crucial for proper cell growth and development (Vousden and Prives, 2009[234]) (Figure 3(Fig. 3)).

Considering the diverse activities of the MDM2 E3 ligase, which enable it to target various proteins, the p53-MDM2 axis can either suppress or promote tumor development, depending on the cellular context and the factors involved. For example, MDM2 can degrade the tumor-promoting factor HIF-1α (hypoxia-inducible factor 1-alpha) in a p53-dependent manner (Ravi et al., 2000[183]). In contrast, it has been demonstrated that mutant p53 (mutp53) drives tumorigenesis by dissociating HIF-1α from MDM2, leading to HIF-1α upregulation (Kamat et al., 2007[98]). Another tumor-suppressive function of MDM2 is its ability to degrade mutp53, thereby stabilizing its levels in cancer cells (García-Cano et al., 2020[59]). Since mutp53 cannot upregulate MDM2 expression, it interacts with other factors, such as heat shock proteins (HSP), valosin-containing protein (VCP), and chaperones like HSP90, to disrupt the mutp53-MDM2 complex, resulting in pro-tumorigenic activities (García-Cano et al., 2020[59]). This dual role of MDM2-as both a tumor suppressor and a tumor promoter-provides new insights into the regulation of mutp53 by the p53-MDM2 interaction (Kadosh et al., 2020[97]). 

Moreover, several MDM2 activities can synergize with or counteract p53 while functioning independently of it. One key role of mitochondrial p53 is regulating mitochondria-mediated apoptosis and mitochondrial respiration during cancer development (Rusin, 2024[190]). Under hypoxic conditions, MDM2 partially relocates to the mitochondria, where it inhibits mitochondrial respiration by reducing the expression of complex I subunit NADH-dehydrogenase 6 (MT-ND6) (García-Cano et al., 2020[59]). This inhibition increases the production of reactive oxygen species (ROS), which subsequently promotes cancer cell migration and invasion (Arena et al., 2018[6]). Conversely, in the cytoplasm, MDM2 binds to the mitochondrial stabilizer NADH ubiquinone oxidoreductase 75 kDa Fe-S protein 1 (NDUFS1), leading to increased ROS generation and the promotion of apoptosis (Elkholi et al., 2019[45]). Despite the growing understanding of MDM2's role in tumorigenesis, particularly through its mitochondrial functions, the precise mechanisms regulating its pro-tumorigenic and anti-tumorigenic activities remain largely unknown. Further research is needed to elucidate how MDM2 mediates these opposing functions within different cellular compartments.

### Role of p53 and MDM2 in cancers

Mutations in p53 have been detected in up to 30 % of all breast cancer cases, and individuals with inherited p53 mutations face an elevated risk of developing ovarian, breast, pancreatic, and colorectal cancers (Lacroix et al., 2006[108]; Muller and Vousden, 2013[148]). While p53 retains its wild-type form in nearly 50 % of tumors, TP53 remains the most frequently mutated gene in human cancers, where p53 function is often impaired or compromised (Herrero et al., 2016[77]; Wasylishen and Lozano, 2016[241]). In parallel, MDM2 overexpression, driven by gene amplification or single nucleotide polymorphisms, is well-established in the tumorigenesis of several human cancers (Bond et al., 2004[16]; Oliner et al., 2016[158]). Importantly, MDM2 overexpression and p53 mutations typically occur as mutually exclusive events, reinforcing the idea that cancer phenotypes are often driven by disruptions in the p53-MDM2 interaction (Oliner et al., 1992[156], 2016[158]; Bond et al., 2004[16]). Additionally, MDM2 contributes to tumorigenesis through other oncogenic activities, including its pro-angiogenic effects, induction of chromosomal instability, degradation of cell cycle regulators, and promotion of epithelial-mesenchymal transition (EMT) via the degradation of E-cadherin (Alt et al., 2005[4]; Miwa et al., 2006[141]; Yang et al., 2006[252], 2008[253]; Bouska et al., 2008[17]; Muthumani et al., 2014[149]; Wolf et al., 2020[244]). Recent research has also linked MDM2 overexpression to resistance against conventional chemotherapy (Hou et al., 2019[81]). For example, p53 gene mutations are a key risk factor for breast cancer (Gasco et al., 2002[60]), where mutant p53 enhances migration, invasion, angiogenesis, scattering, stem cell proliferation, survival, and tissue remodeling. The role of p53 in breast cancer progression is complex and multifaceted, with p53 interacting with other signaling pathways, including the Wnt/β-catenin and PI3K/Akt/mTOR pathways, to modulate breast cancer cell behavior (Reddy et al., 2020[185]; Shahcheraghi et al., 2020[194]). Furthermore, p53 mutations are also an important risk factor in hepatocellular carcinoma (HCC). Epidemiological studies have shown that chronic viral infections (such as HBV, HCV, and HIV) and metabolic disorders (such as metabolic syndrome) are associated with disruption of the MDM2-p53 axis in HCC (Cao et al., 2020[24]). Hepatitis viruses employ multiple mechanisms to persist in hepatocytes, including inducing p53 mutations, silencing or overexpressing MDM2, stabilizing MDM2 levels, and accelerating p53 degradation. These processes trigger various stress responses, including oxidative stress, energy metabolism shifts, chronic ER stress, genetic instability, and abnormal antitumor gene expression, ultimately driving the transformation of hepatocytes into hepatoma cells (Cao et al., 2020[24]). The MDM2-p53 interaction has also been implicated in the regulation of glucolipid metabolism in the liver, where its dysregulation contributes to metabolic diseases such as metabolic syndrome and non-alcoholic fatty liver disease (NAFLD), conditions that can progress to HCC under certain circumstances (Guillen-Sacoto et al., 2017[74]). Studies suggest that lipid accumulation in hepatocytes leads to the overexpression of microRNA-21, which enhances the expression of carcinogenesis-related proteins (CCNB1, CCND1, and SREBP1C) by inhibiting p53. MicroRNA-21 targets HBP1, a transcriptional activator of p53, and its overexpression drives G_1_/S and G_2_/S transitions in hepatocytes, promoting de novo lipogenesis by modulating the HBP1-p53 axis. Conversely, knocking down microRNA-21 inhibits the G_1_/S transition and suppresses hepatoma proliferation (Wu et al., 2016[245]).

### The disruption of p53-MDM2 interaction in cancers

The dysregulation of the p53-MDM2 feedback loop is one of the most well-documented disruptions in cancer, often manifesting as overexpression of MDM2 and/or p53. This phenomenon is linked to the activation of stress-induced p53 pathways, which fail to adequately induce growth arrest and/or apoptosis (Klein and Vassilev, 2004[103]). Additionally, the impaired activation of various oncogenes, such as Myc, Ras, E2F-1, and β-catenin, has been shown to enhance ARF activation, a known MDM2 suppressor, which negatively modulates MDM2 function (Eischen et al., 1999[44]; Manfredi, 2010[131]; Hu et al., 2012[86]). As a result, activated p53 exerts tumor-suppressive effects in this context. ARF promotes the sequestration of MDM2 in the nucleolus, reducing its E3 ubiquitin ligase activity and ultimately segregating MDM2, thereby diminishing its negative regulatory effect on p53 (Weber et al., 1999[242]; Zhang and Xiong, 1999[268]). Disruptions in the ARF/MDM2/p53 signaling pathway are frequently observed in various cancer types (Sherr, 2006[201]). Other oncogenes, such as AKT and Wip1, directly regulate MDM2. The IGF-1/AKT oncogenic pathway is closely linked to cell fate and proliferation, where AKT kinase phosphorylates MDM2 at Ser-166 and Ser-186, leading to downregulation of p53 activity (Grossman et al., 1998[71]; Zhou et al., 2001[273]). Wip1, an oncogenic serine/threonine phosphatase induced and regulated by p53 following genotoxic stress, dephosphorylates MDM2 at Ser-395, restoring MDM2 activity and maintaining p53 at steady-state levels, thus ensuring proper regulation of the p53-MDM2 feedback loop. Notably, Wip1 is frequently overexpressed in various human cancers, indicating a disruption of the p53-MDM2 interaction (Peuget et al., 2024[162]). To date, no candidate drugs targeting the MDM2-p53 interaction have been successfully validated in pharmacological stages (preclinical/clinical trials) as cancer therapies. This can be attributed to the low efficacy and high toxicity of the tested prodrugs in experimental models. Understanding these challenges is crucial for making informed decisions and advancing the field of drug development (Zanjirband and Rahgozar, 2019[259]; Mullard, 2020[147]). The accumulated data suggest that a deeper understanding of the complexity and intricacy of the p53-MDM2 interaction is important for gaining valuable insights into cancer therapy. The complex biology and regulation of this interaction, along with its implications in cancer, are summarized in Table 1(Tab. 1) (References in Table 1: Asahara et al., 2003[7]; Dar et al., 2013[37]; de Toledo et al., 2000[38]; Farkas et al., 2021[50]; Fischer, 2017[52]; Fu et al., 2023[55]; Hafner et al., 2017[75]; Levine, 2020[113]; Lin et al., 2024[123]; Maya et al., 2001[133]; Menendez et al., 2009[136]; Meulmeester et al., 2005[139]; Mullard, 2020[147]; Peuget et al., 2024[162]; Rayburn et al., 2009[184]; Sammons et al., 2020[192]; Thomasova et al., 2012[221]; Vlatkovic et al., 2000[231]; Vousden and Prives, 2009[234]; Yao et al., 2024[256]; Zafar et al., 2023[258]; Zanjirband and Rahgozar, 2019[259]; Zhang et al., 2013[262]).

## Mechanistic Insights into Natural Products Targeting the p53-MDM2 Pathway

**Figure 4:**
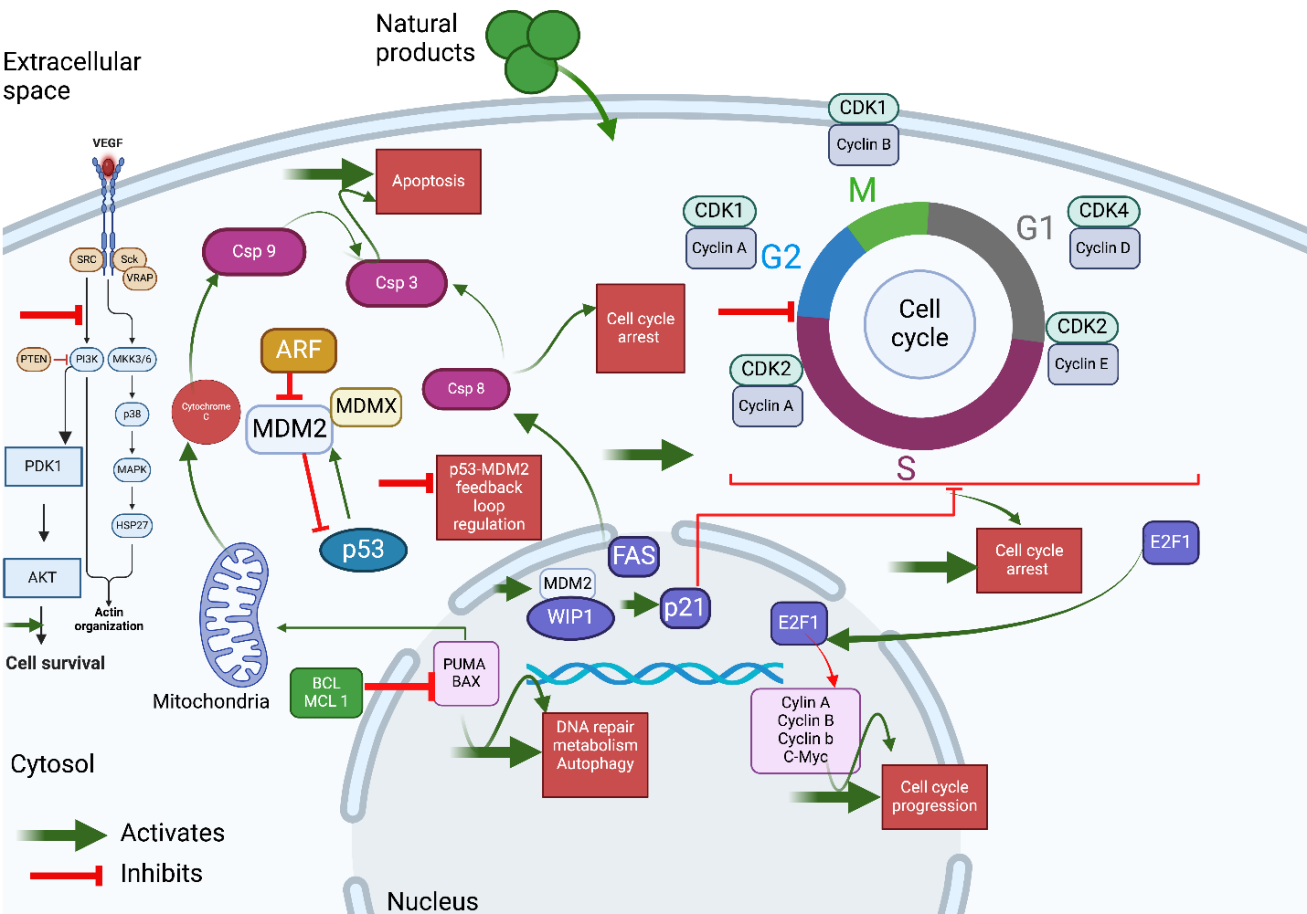
The impact of natural products on the cellular processes and target genes mediated by p53-MDM2 interaction. Crosstalk of the anticancer activity of natural products mediated by the p53-MDM2 pathway: When the MDM2-p53 interaction is modulated by natural products, p53 accumulates and activates its direct transcriptional targets, leading to protein synthesis. This results in various cellular responses: p21 induces cell cycle arrest; PUMA, NOXA, and BAX activate the intrinsic apoptotic pathway; FAS induces the extrinsic apoptotic pathway; MDM2 and WIP1 regulate p53 feedback and other pathways involved in DNA repair and cell metabolism. Cell cycle progression is primarily regulated by p53 activity via the p21 protein, which binds to and inhibits the CDK/cyclin complexes, blocking cell cycle progression. Consequently, CDK4/6 with cyclin D/E mediates the activity of RB and E2F1. This activation eventually releases E2F1, which then activates its transcriptional program, leading to cell cycle progression. Key Proteins and Pathways: AKT: Ak mouse strain thymoma protein*, *PTEN: Phosphatase and TENsin homolog, PI3K: phosphoinositide 3-kinase, MAPK: Mitogen-activated protein kinase, HSP27: heat shock protein 27, SRC: Proto-oncogene tyrosine-protein kinase, SCK: Shc-related adaptor protein, MKK3/6: Map kinase kinase isoforms 3 and 6, VRAP:VEGF-receptor-associated protein/T-cell-specific adaptor molecule, PDK1: Phosphoinositide-dependent kinase-1, Csp9: Caspase-9, Csp8: Caspase-8, Csp3: Caspase-3, Arf: ADP-ribosylation factor, MDM2: The murine double minute 2, MDMX: The murine double minute X, p53: Tumor suppressor 53, BCL: B-cell lymphoma 2 protein, MCL: Induced myeloid leukemia cell differentiation protein, PUMA: P53 upregulated modulator of apoptosis, BAX: Bcl-2-associated X protein, Wip1: Wild-type p53-induced phosphatase1, p21: cyclin-dependent kinase inhibitor 1, E2F1: E2F transcription factor 1, FAS: Fas cell surface death receptor, C-Myc: C-MYC proto-oncogene, BHLH transcription factor protein, CDK1: cyclin-dependent kinase 1, CDK2: cyclin-dependent kinase 2, CDK4: cyclin-dependent kinase 4

Bioactive natural compounds are well-known for producing a wide array of secondary metabolites with diverse structures, which have played a pivotal role in the development of approximately 50 % of anti-cancer drugs over the past several decades (Kamath et al., 2023[99]; Chaachouay and Zidane, 2024[27]; Chaudhry et al., 2024[29]; Chunarkar-Patil et al., 2024[36]; Ijaz et al., 2024[89]; Nandi et al., 2024[151]). Among these natural molecules, several have been reported to target the p53-MDM2 interaction and can be classified into three categories:

a) Direct inhibitors of MDM2 expression and/or protein stability: These compounds reduce MDM2 levels by inhibiting its expression or destabilizing the protein, thereby enhancing p53 activity. Examples include: i) Curcumin: Downregulates MDM2 expression via the PI3K/mTOR/ETS2 pathway, leading to reduced MDM2 levels and increased p53 activity (Sultana et al., 2021[213]); ii) Resveratrol: Acts as a direct inhibitor of MDM2 expression and prevents MDM2-mediated p53 degradation, thereby promoting p53 stability (Merlin et al., 2021[137]); iii) Gambogic Acid: Inhibits MDM2 by downregulating its expression, stabilizing p53, and inducing apoptosis in cancer cells (Foggetti et al., 2017[53]).

b) Inhibitors of the p53-MDM2 binding and activators of wild-type p53: These compounds disrupt the p53-MDM2 interaction, thereby reactivating wild-type p53. Examples include: i) Nutlin-3: A potent MDM2 antagonist that inhibits the p53-MDM2 interaction, leading to the activation of p53 (Lerma Clavero et al., 2023[112]); ii) Epigallocatechin Gallate (EGCG): Disrupts p53-MDM2 binding, preventing p53 degradation and enhancing its accumulation in cells (Bahena Culhuac and Bello, 2024[11]); iii) Leucomalachite Green (LMG): Inhibits the binding of p53 to MDM2, reactivating p53 in cancer cells (Koo et al., 2022[105]).

c) Inhibitors of MDM2's E3 ligase activity, stabilizing p53: These compounds inhibit MDM2's E3 ligase activity, preventing the ubiquitination and degradation of p53, thus stabilizing and activating p53. Examples include: i) MI-219: Blocks MDM2's E3 ligase activity, stabilizing p53 and enhancing its tumor-suppressive functions (Yang et al., 2021[255]); ii) Lithocholic Acid: Inhibits MDM2's E3 ligase function, leading to increased stability and activity of p53 (Yao et al., 2024[256]); iii) Oridonin: A diterpenoid that inhibits MDM2's E3 ligase activity, resulting in the stabilization and accumulation of p53 (Zhu et al., 2019[276]) (Figure 4(Fig. 4)).

This section will explore the data on natural compounds that modulate the p53-MDM2 interaction, focusing on their structural aspects, binding modes, and mechanisms of action (Table 1(Tab. 1)). 

### Polyphenols

Flavonoids, a significant group of over 10,000 secondary metabolites (Ullah et al., 2020[226]), ave garnered considerable attention for their ability to counteract free radicals, modulate cellular metabolism, and mitigate oxidative stress associated with several severe diseases, including Parkinson's, Alzheimer's, cardiovascular conditions, and cancer (Vazhappilly et al., 2019[228]; Giordo et al., 2021[69], 2022[68]; Sharifi-Rad et al., 2022[199]; Shaito et al., 2023[195]). Numerous studies suggest that natural flavonoids exhibit potent anticancer activities through various mechanisms, including the inhibition of MDM2 expression (Merlin et al., 2021[137]). Genistein (4′,5,7-trihydroxyisoflavone), a natural isoflavone abundantly found in soybeans, has been shown to downregulate MDM2 at both transcriptional and post-translational levels (Bhat et al., 2021[14]). *In vitro* chemopreventive studies in several human cancer cell lines revealed that genistein decreased MDM2 expression levels independently of p53. It also inhibited the tyrosine kinase pathway regulating MDM2, while simultaneously increasing p21 levels. *In vivo* studies further confirmed genistein's antitumor activity, which is related to its inhibitory effects on MDM2 expression (Tuli et al., 2019[224]; Gao et al., 2020[58]). Gao et al. (2020[58]) demonstrated that prolonged genistein treatment significantly reduces epidermal growth factor receptor (EGFR) expression and moderates downstream signaling molecules (JAK1/2, MDM2, STAT3, and Akt phosphorylation), leading to the inhibition of the JAK1/2-STAT3 and AKT/MDM2/p53 pathways. This ultimately results in apoptosis, cell cycle arrest, and reduced proliferation of esophageal carcinoma cells (Gao et al., 2020[58]).

Apigenin (4′,5,7-trihydroxyflavone) is a well-known flavone widely distributed in nuts, fruits, vegetables, and herbs. Its low intrinsic toxicity (Tang et al., 2017[217]), combined with its potent effects on cancer cell growth (Yan et al., 2017[251]), survival (Rahmani et al., 2022[178]), or apoptosis (Shukla and Gupta, 2008[205]) has drawn significant interest. A study by Fang et al. (2005[48]) demonstrated that apigenin attenuates angiogenesis and tumor growth by enhancing p53 activity through AKT-mediated phosphorylation of its negative regulator HDM2 in ovarian cancer cells (Fang et al., 2005[48]). The study also indicated that apigenin's effect on HDM2 downregulation is mediated by the PI3K/Akt pathway (Fang et al., 2005[48]). Furthermore, apigenin has been shown to stabilize p53 activation and inhibit metastasis (Sherr, 1998[202]; Zheng et al., 2005[273]).

Quercetin (3,5,7,3′,4′-pentahydroxyflavone), one of the most extensively studied flavonols, is found in fruits, tea, wine, vegetables, and other plants (Hossain et al., 2022[80], Aghababaei and Hadidi, 2023[1]). Research indicates that reactive oxygen species (ROS) activate p53 via upstream signal transduction, promoting programmed cell death in abnormal cells (Asgharian et al., 2022[8]). Quercetin has been shown to inhibit tumor cell proliferation by stimulating p53 and NF-κB (Vidya Priyadarsini et al., 2010[230]). An *in vitro* study on human leukemia cells demonstrated that quercetin enhances p53 phosphorylation and induces apoptosis in a dose-dependent manner (Mertens-Talcott et al., 2005[138]). Similarly, Tanigawa et al. (2008[219]) found that quercetin increases p53 phosphorylation without upregulating its transcription (Tanigawa et al., 2008[219]). In another study, quercetin accelerated apoptosis and growth arrest in wild-type p53-containing A549 human lung cancer cells (Chan et al., 2013[28]). Quercetin also induced apoptosis in glioblastoma cells by upregulating MDM2 mRNA expression, activating caspase-3, and decreasing p53 levels, affecting the regulation of the MDM2-p53 axis (Wang et al., 2014[236]) Molecular dynamics studies revealed that quercetin binds to the MDM2-p53 hydrophobic groove, altering its conformation and disrupting the MDM2-p53 interaction through π-π stacking between MDM2's Tyr 51 and quercetin (Verma et al., 2013[229]). Furthermore, Yang et al. (2016[254]) demonstrated that quercetin reduces cell viability, triggers apoptosis, and induces cell cycle arrest in HT-29 cells by inhibiting the Akt-CSN6-Myc axis, another pathway regulating the MDM2-p53 interaction (Zhou et al., 2001[274]; Zhao et al., 2011[272]; Yang et al., 2016[254]).

Epigallocatechin gallate (3',4',5,5',7-pentahydroxy 3-gallic acid flavane), (EGCG), the main catechin in green tea, has potent antioxidant and anticancer properties (Johnson et al., 2012[95]). EGCG treatment inhibits anchorage-independent growth in human lung cancer cells by stabilizing p53, promoting its nuclear localization, and reducing MDM2 nuclear accumulation. EGCG also enhances p53 phosphorylation at Ser15 and Ser20, thereby increasing its transcriptional activity. This compound likely promotes MDM2 expression in a p53-dependent manner, preventing the ubiquitination of p53 by MDM2 (Jin et al., 2013[94]). A study using NMR, atomistic simulation, AUC, and SAXS analyses identified p53's N-terminal domain (NTD) as the primary binding site for EGCG, which interrupts the p53-MDM2 interaction and stabilizes p53 by inhibiting its ubiquitination and degradation (Zhao et al., 2021[271]).

Oroxylin A (5,7-dihydroxy-6-methoxyflavone), a natural flavone from Oroxylum and Scutellaria species, has been shown to induce apoptosis in HepG2 hepatocellular carcinoma cells by stabilizing p53 at the post-translational level through the downregulation of MDM2 and inhibition of its E3 ligase activity (Mu et al., 2009[145]).

Involucrasin A, a recently discovered natural flavanone from Shuteria involucrata, has demonstrated significant anticancer effects in colon cancer cells (HCT-116) by inhibiting the phosphorylation of Akt and MDM2, which leads to elevated p53 levels (Wei et al., 2023[243]).

Chrysin (5,7-dihydroxyflavone) and wogonin (5,7-dihydroxy 8-methoxyflavone) are similar flavones. TRAIL is a promising antitumor agent that inhibits various tumor cell growth without causing any damage to the peripheral normal tissues (Ding et al., 2012[42]). However, several cancers remain resistant to TRAIL, including TRAIL-resistant human T-cell leukemia virus type 1 (HTLV-1) and Adult T-cell leukemia/lymphoma (ATL) cells. Ding et al. (2012[42]) demonstrated that both chrysin and wogonin inhibit the p53 antagonist MDM2 by increasing p53 levels and upregulating TRAIL-R2. TRAIL-R2 encodes the receptor responsible for the expression of TRAIL protein, a key target gene of p53. This mechanism successfully overcame TRAIL resistance in HTLV-1-associated ATL cells by downregulating the anti-apoptotic FLICE-inhibitory protein (c-FLIP), which is a key inhibitor of the death receptor signaling pathway and blocks caspase 8 activation (Ding et al., 2012[42]).

Tricetin (3′,4′,5′,5,7-pentahydroxyflavone) is a multi-hydroxylated flavone found in certain medicinal plants (Wu et al., 2022[246]). In studies on MCF-7 breast cancer cells, tricetin inhibited cell growth by arresting the cell cycle in the G_2_/M phase and inducing apoptosis. This was associated with the activation of ATM, which phosphorylates p53 at Ser15, leading to increased p53 stability and reduced MDM2-p53 interaction (Hsu et al., 2009[84]).

Hinokiflavone, a natural bioflavonoid with potent anticancer properties (Patel, 2024[160]), was investigated by Zhang et al. (2022[267]), who concluded that it suppresses MDM2 mRNA synthesis at the transcriptional level. This inhibition results in increased p53 expression, activation of the p53 pathway, and reduced survival of HCT116 colon cancer cells via apoptosis induction and G_2_/M phase arrest (Zhang et al., 2022[267]).

Curcumin, a dietary polyphenol derived from Curcuma species, is renowned for its biological properties, particularly its anti-inflammatory and anti-angiogenic effects (Quispe et al., 2022[176]; Azzini et al., 2024[10]). To explore how curcumin influences gene expression and carcinogenesis, Li et al. (2007[119]) investigated its impact on various cancer cell lines, including prostate cancer LNCaP (p53 wild type), breast cancer MCF-7 (p53 wild type and p53 knockout), and PC3 (p53 null). Their findings revealed that curcumin inhibits MDM2 expression in a dose-dependent manner, with inhibition occurring at the transcriptional level and affecting MDM2 promoter activity (Li et al., 2007[119]). Further *in vitro* and *in vivo* studies showed that curcumin downregulates MDM2 expression in both p53-wild-type and p53-null prostate cancer cells by inhibiting the PI3K/mTOR/ETS2 pathway (Li et al., 2007[119]). Additionally, curcumin induces apoptosis through cell cycle arrest by upregulating the expression of p27, p21, and p16, increasing ER stress, and reducing MDM2 levels (Srivastava et al., 2007[208]; Rivera et al., 2017[187]). A similar study on multiple myeloma RPMI 8226 cells demonstrated that curcumin downregulates MDM2 expression while upregulating p53 and Bax expression (Li et al., 2015[122]). According to Patiño-Morales et al., curcumin stabilizes and extends the active period of p53 by enhancing its interaction with NAD(P)H quinone oxidoreductase 1 (NQO1), ultimately leading to cervical cancer cell death *in vitro* (Patiño-Morales et al., 2020[161]). Interestingly, curcumin was found to be less effective against breast cancer cell lines compared to cervical cancer cells, likely due to the presence of wild-type p53 in cervical cancer cells, whereas breast cancer cells often contain mutated p53 (Patiño-Morales et al., 2020[161]).

Resveratrol, a natural stilbene monomer, possesses potent antioxidant, anti-inflammatory, neuroprotective, vasculoprotective, and anticancer properties (Giordo et al., 2020[65], 2021[66], 2022[68]; Ramli et al., 2023[179]). The report by She et al. was the first to demonstrate the effect of resveratrol on p53 increase in epidermal JB6 cells, especially in the phosphorylated state (She et al., 2001[200]). In p53-positive Hep G2 cells, resveratrol inhibited cell growth by inducing p53-activated apoptosis. Additionally, resveratrol caused cell cycle arrest in the G_1_ phase and concurrently upregulated p21 protein expression ((Kuo et al., 2002[107]). Resveratrol was also found to activate the ERK and/or p38 kinase pathways, which promote p53 activation, induce cell cycle arrest, and facilitate DNA repair (Hsieh et al., 2011[83]). Furthermore, resveratrol influenced p53-mediated mitochondrial functions (Delmas et al., 2011[39]). 

A study by Ferraz da Costa et al. (2012[51]) reported that resveratrol increased p53 levels in MCF-7 cells without affecting its transcriptional activity. Moreover, transient transfection of wild-type p53 into p53-negative H1299 cells dramatically enhanced susceptibility to apoptosis in resveratrol-treated cells (Ferraz da Costa et al., 2012[51]). Resveratrol also inhibited the viability of CO115, HCT116, and SW480 cells while upregulating p53 and its target genes, including PUMA and Bax (Liu et al., 2019[126]). Bioinformatics analysis further revealed that resveratrol elevated p53 expression in a dose-dependent manner by inhibiting p-Akt and p-MDM2 signaling (Fan et al., 2020[47]). Another study demonstrated that resveratrol preserved AEC₂ cell integrity by activating Sirt1 expression, promoting p53 instability, and stimulating the phosphorylation of both Akt and MDM2 (Navarro et al., 2017[153]). However, resveratrol at concentrations greater than 10 µM was shown to downregulate Sirt1 expression, inhibit cellular plasticity, and induce apoptosis. This effect was accompanied by simultaneous acetylation of p53 in CRC cells, prompting the activation of p53, p21, Bax, and cytochrome C, as well as cleavage of caspase-3 (Brockmueller et al., 2023[21]). 

Gossypol, a naturally occurring phytochemical derived from cotton plants (Gossypium species), appears to be a promising anticancer agent (Stein et al., 1992[210]). One study showed that the viability of LAPC4, PC3, and DU145 cancer cells was reduced through the induction of DNA damage and activation of p53 (Volate et al., 2010[233]). Xiong et al. (2017[248]) also reported gossypol's ability to inhibit both VEGF and MDM2 expression in human breast cancer cells, irrespective of whether p53 was mutant or wild-type (Xiong et al., 2017[248]). 

Gambogic acid, a naturally prenylated xanthone, was found to suppress tumor growth by inhibiting MDM2 expression while promoting p53 activation (Gu et al., 2008[72]). A related study demonstrated that gambogic acid inhibits Bcl-2 expression in MCF-7 cells by increasing p53 levels, ultimately inducing cell death (Zhai et al., 2008[260]). Further investigation identified a negative correlation between p53 activation and the promotion of p21^Waf1/CIP1^ expression, which enhances apoptosis in gambogic acid-treated MCF-7 cells via suppression of MDM2 (Rong et al., 2009[189]). A parallel molecular docking study supported these findings, indicating that gambogic acid binds directly to MDM2, functioning as a direct MDM2 inhibitor (Leão et al., 2013[110]).

### Terpenoids

With more than 80,000 structures discovered, terpenoids, also known as terpenes or isoprenoids, represent the most prominent family of natural products in all living organisms (Christianson, 2017[35]). They are essential for supporting human health and have been employed as antioxidant, anti-inflammatory, anti-aggregator, anticoagulant, anticancer, antimicrobial, neuroprotective, sedative, anti-allergic, and analgesic agents (Zhao et al., 2016[270]). Numerous studies have highlighted the potent anticancer properties of natural terpenoids, particularly their ability to inhibit MDM2 expression through regulation of p53 levels.

Using a structure-based computational screening method designed to identify molecules that specifically target MDM2, Qin and collaborators identified three natural dimeric sesquiterpene lactones from Inula japonica-namely japonicone A, inulanolide A, and lineariifolianoid A-as potent inhibitors of MDM2 expression in breast cancer cells. Japonicone A was shown to inhibit cell growth, reduce cell proliferation, and induce apoptosis and G₂/M phase cell cycle arrest via an MDM2-dependent mechanism, independent of p53 status. Moreover, no toxicity was observed in breast cancer xenograft models treated with japonicone A, which effectively inhibited tumor growth and lung metastasis (Qin et al., 2015[174]). Inulanolide A underwent both *in vitro* and *in vivo* anticancer experiments, demonstrating its dual inhibitory effects on MDM2 and NFAT1 in breast cancer cells. This anticancer activity was selective in both p53-dependent and p53-independent manners, leading to apoptosis induction, reduced cell proliferation, and G₂/M phase arrest. Furthermore, a reduction in MDM2, NFAT1, and cell proliferation-related proteins was observed, alongside an increase in apoptosis-related proteins (Qin et al., 2016[173]). Another study examined the anti-tumorigenic effects of inulanolide A in prostate cancer, showing its ability to inhibit migration, invasion, and proliferation of prostate cancer cells, regardless of androgen receptor (AR) responsiveness and p53 status. This study demonstrated a high affinity for binding to the RING domains of both MDM2 and MDMX proteins (Qin et al., 2017[172]). A similar study on breast cancer revealed that lineariifolianoid A significantly influenced apoptosis, cell cycle progression, proliferation, and colony formation in MCF7 and MDA-MB-231 cells in a dose-dependent and p53-independent manner (Jiang-Jiang et al., 2016[93]). Another sesquiterpene lactone, parthenolide, isolated from Tanacetum parthenium, was reported to induce ATM-dependent MDM2 ubiquitination and proteasomal degradation, leading to p53 activation and the activation of other tumor suppressors that regulate MDM2 (Nasim and Crooks, 2008[152]; Gopal et al., 2009[70]).

Several reports have demonstrated the potent anticancer activity of natural diterpenoids, particularly triptolide, which is extracted from the Chinese plant Tripterygium wilfordii. This diterpene has shown potent antitumor activity against various cancer cells through different mechanisms (Huang et al., 2012[88]; Tamgue and Lei, 2017[216]). One study revealed that ionizing radiation-resistant (IR-resistant) acute lymphoblastic leukemia (ALL) cells are sensitive to triptolide, which reversed IR resistance in ALL cells by inducing an MDM2-overexpressing phenotype. The accumulation and activation of p53-induced by many chemotherapeutic drugs that kill cancer cells through DNA damage and cellular stress-lead to increased p53 activation, which subsequently induces MDM2 expression. Inhibition of p53 and induction of XIAP are key mechanisms involved in the development of IR- or chemo-resistance in wild-type p53/MDM2-overexpressing ALL cells (Huang et al., 2013[87]). To determine whether the effect of triptolide on MDM2 expression is p53-dependent, Xiong and colleagues investigated its impact on paired MDA-MB-468 (p53 mutant) and MCF-7 (wild-type p53) cell lines. Their results showed that triptolide inhibited MDM2 protein expression in a time- and dose-dependent manner, while increasing p53 accumulation without activating its function. Thus, the inhibitory effect of triptolide on MDM2 mRNA and protein expression was independent of p53 status (Xiong et al., 2017[248]). Epoxy clerodane diterpene, isolated from the stems of Tinospora cordifolia, has been found to exhibit remarkable anticancer effects (Dhanasekaran et al., 2009[40]). A study led by Subash-Babu and collaborators on the antitumor activity of epoxy clerodane diterpene against MCF-7 cells demonstrated its ability to upregulate Cdkn2A, pRb1, and p53 proteins, while simultaneously downregulating MDM2. The increase in p53 expression activated the Bax apoptotic pathway, contributing to the suppression of MDM2 expression (Subash-Babu et al., 2017[211]). Another bioactive diterpene, oridonin, derived from the traditional Chinese herb Rabdosia rubescens, exhibits a wide range of biological activities, particularly anticancer, antibacterial, and anti-inflammatory effects (Xu et al., 2018[250]). A study by Zhu et al. (2019[276]) reported that oridonin stimulates p53-mediated cell cycle arrest and apoptosis in neuroblastoma cells by promoting the cleavage of MDM2-p60 (Zhu et al., 2019[276]). 

Studies on the ortho-diphenolic diterpene carnosol, found in sage (Salvia officinalis) and rosemary (Rosmarinus officinalis), have demonstrated its potent antioxidant and anticancer effects (O'Neill et al., 2020[154]). *In vitro* anticancer studies using the U87MG human glioblastoma cell line model revealed that carnosol modulates cellular proliferation by elevating intracellular p53 levels. This was achieved by promoting the transcriptional reactivation of p53, disrupting the p53-MDM2 interaction, and inducing cell cycle arrest and apoptosis (Giacomelli et al., 2016[62]). Continuing their research, Giacomelli and collaborators reported that carnosol decreases CD44 gene expression. This effect is associated with inhibition of the MDM2-p53 complex and the subsequent increase in intracellular p53 levels (Giacomelli et al., 2017[61]).

Ginsenosides, a group of steroid glycosides and triterpene saponins found exclusively in the roots of Panax ginseng, a highly renowned herb in traditional Asian medicine, have demonstrated strong anticancer activity in both *in vitro* and *in vivo* studies. Compounds such as 20(R)-dammarane-3β,12β,20,25-tetrol and 20(S)-25-methoxyldammarane-3β,12β,20-triol (also known as 25-OH-PPD and 25-OCH₃-PPD, respectively) have shown potent anti-prostate cancer effects by regulating cell proliferation, apoptosis, cell cycle progression, and tumor growth. 25-OH-PPD also decreased MDM2 levels without affecting p53 expression, decreased cell survival, suppressed proliferation, and triggered apoptosis, leading to G_1_ cell cycle arrest in both LNCaP and PC3 cells (Wang et al., 2008[239]). Furthermore, 25-OCH₃-PPD was found to decrease the levels of cyclin D1, CDK2, E2F1, and MDM2 while increasing or activating cleaved caspase-3, -8, -9, and cleaved PARP (Wang et al., 2008[238]). Similar results were observed in breast cancer cells, where 25-OCH₃-PPD downregulated MDM2 expression at both transcriptional and posttranslational levels in a time- and dose-dependent manner, irrespective of p53 status (Wang et al., 2012[240]). A study on the antiproliferative and pro-apoptotic effects of 20(S)-ginsenoside Rg3 in MDA-MB-231 cells demonstrated that it reduced mutant p53 levels in both a concentration- and time-dependent fashion. Concurrently, 20(S)-ginsenoside Rg3 increased the association of MDM2 with p53 in these cells (Kim et al., 2014[101]). Another study reported that the survival of NOZ and GBC-SD gallbladder cancer cells was inhibited in a dose-dependent manner by 20(S)-ginsenoside Rg3. This inhibition was achieved through G₁ phase arrest, promoting senescence and apoptosis by inhibiting MDM2 levels, leading to the accumulation of p53 and p21 1 (Zhang et al., 2015[261]).

Ganoderic acids, a group of triterpenes isolated from Ganoderma mushrooms, have been shown to possess various biological activities, including antitumor properties (Kimura et al., 2002[102]). Bin et al. demonstrated that ganoderic acid A has an inhibitory effect on LNCaP prostate cancer cells in a concentration-dependent manner by promoting p53-mediated apoptosis (Bin et al., 2019[15]). A previous virtual screening study of Ganoderma lucidum triterpenoids predicted a strong binding affinity of ganoderic acid A for MDM2 (Froufe et al., 2013[54]). Recent research confirmed these findings, showing that ganoderic acid and its amide derivatives regulate the MDM2-p53 pathway in MCF-7 cells (Jia et al., 2023[92]). Chen et al. demonstrated that ganoderic acid T promotes cell aggregation, suppresses cell migration, and inhibits cell adhesion in HCT-116 human colon cancer cells in a concentration-dependent manner, highlighting the important role of p53 in its anti-invasion effects (Chen and Zhong, 2011[31]). A study on the cytotoxicity and cell cycle arrest capabilities of ganoderic acid against highly metastatic human colon tumor HCT-116 cells, p53-null lung cancer H1299 cells, and lung cancer 95-D cells demonstrated remarkable effects in both a concentration- and time-dependent manner. In 95-D and HCT-116 p53^+/+^ cells, the cell cycle was arrested at the G₁ phase, while in H1299 and HCT-116 p53^−/−^ cells, ganoderic acid was able to arrest the cell cycle in the S phase or at the G₁/S transition. Based on these findings, Chen and Zhong suggested that ganoderic acid may target p53 (Chen and Zhong, 2009[30]). Aqueous and methanol extracts from Ganoderma lucidum have been shown to inhibit interleukin-3-dependent lymphoma cell (DA-1) proliferation (Calviño et al., 2011[23]). Western blot analysis revealed that the aqueous extracts elevated Bax levels after 13 hours, as well as p53 and Mdm2 levels after 19 hours, with a subsequent reduction in all these proteins at 24 hours. Similarly, the methanol extract increased p53 and Mdm2 levels at 19 hours, followed by a decrease at 24 hours (Calviño et al., 2011[23]).

Cucurbitacins are another class of terpenoids with notable anticancer activity (Attar et al., 2022[9]). Among the more than 10 groups of cucurbitacins, the most commonly isolated and studied are A, B, E, and I (Attar et al., 2022[9]). Zhou et al. demonstrated that cucurbitacin B inhibits the proliferation of benign prostatic hyperplasia epithelial cell line (BPH-1). Molecular analysis showed that cucurbitacin B increased the mRNA levels of MDM2 and thrombospondin 1 (THBS1). Immunocytochemistry results further indicated that cucurbitacin B treatment elevated the protein expressions of p53 and MDM2 (Zhou et al., 2023[275]).

An in vitro and in vivo study on the triterpenoid saponin platycodin D demonstrated its ability to inhibit cell growth in human breast cancer MDA-MB-231 cells by suppressing MDM2 and MDMX, and by reducing mutant p53 expression levels (Kong et al., 2016[104]). Another study found that platycodin D treatment induced apoptosis in MDA-MB-231 cells by upregulating PUMA, a modulator of p53-mediated apoptosis (Chen et al., 2022[33]).

An in silico study reported the binding affinity of fucoxanthin to the p53 gene, CDK2, and tubulin (Indra Januar et al., 2012[90]). Wang et al. (2014[237]) evaluated the anticancer activity of fucoxanthin against the human bladder cancer T24 cell line and revealed its inhibitory effects on both cell growth and colony formation. Additionally, fucoxanthin was able to induce apoptosis and G₀/G₁ phase cell cycle arrest by suppressing the mortalin-p53 complex and reactivating p53 (Wang et al., 2014[237]).

Several in silico screening studies have identified new MDM2-p53 inhibitors, including lithocholic acid, which demonstrated dual inhibitory activity against both MDMX-p53 and MDM2-p53 interactions. Another study showed that lithocholic acid induced apoptosis in wild-type p53 HCT116 cells in vitro (Vogel et al., 2012[232]). Similarly, Muhseen and Li reported the strong binding affinity of 3-trans-p-coumaroyl maslinic acid, betulonic acid, and silvestrol to the active site of MDM2, comparable to the binding affinity exhibited by Nutlin-3a, a known inhibitor of the p53-MDM2 interaction. Results suggest that these compounds occupied the p53 binding regions of MDM2, thereby inhibiting the p53-MDM2 interaction (Muhseen and Li, 2019[146]). Comparable results were found in a recent study, which demonstrated the strong binding affinity of three compounds-justin A, 6-hydroxy justicidin A, and 6′-hydroxy justicidin B-at the active site of MDM2, surpassing the binding affinity of Nutlin-3a (Shoaib et al., 2023[204]).

### Alkaloids

Despite the relatively low number of alkaloids identified from plants (approximately 3,000 molecules), many of them are considered potent anticancer agents. Numerous reports have demonstrated the ability of alkaloids to induce self-ubiquitination and degradation of MDM2 by disrupting the MDM2-DAXX-HAUSP interactions (Dhyani et al., 2022[41]). The natural isoquinoline alkaloid berberine has been shown to downregulate the MDM2 oncoprotein in wild-type p53 acute lymphoblastic leukemia (ALL) cell lines, leading to the induction of apoptosis (Zhang et al., 2010[263]). A similar study confirmed that the berberine-induced downregulation of MDM2 expression also reduced XIAP levels, promoting apoptosis in ALL cells independent of p53 status (Liu et al., 2013[124]). 

Another alkaloid, matrine, has been reported to inhibit MDM2 expression by reducing MDM2 mRNA synthesis in liver cancer cells. Additionally, matrine sensitizes MDM2-overexpressing liver cancers to etoposide-induced apoptosis, independent of p53 levels. The monoamine alkaloid melatonin has been found to inhibit MDM2 transcription and post-transcriptional expression, reduce MDM2 phosphorylation, and promote p53 acetylation, resulting in p53 activation in MCF-7 cells (Proietti et al., 2014[171]). In human gastric cancer cells, melatonin induced cell cycle arrest and downregulated CDC25A, phospho-CDC25A, and p21. Moreover, melatonin upregulated Bax, downregulated Bcl-xL, activated caspase-3, and increased levels of cleaved caspase-9. Melatonin also increased p53 levels by inhibiting MDM2 phosphorylation at Ser166 and Akt phosphorylation at Thr308 (Song et al., 2018[207]). Another study reported that melatonin upregulated the Nrf2 signaling pathway by activating the MDM2-p53-p21 signaling cascade (Tao et al., 2022[220]). 

Overall, significant progress has been made in studying natural products that target the p53-MDM2 interaction for cancer prevention and therapy. However, there remains an urgent need to address key challenges related to in vivo efficacy, bioavailability, potential toxicity, and mechanisms of action in clinical models to develop efficient and safe preventive therapies. A comprehensive summary of the aforementioned natural products and their anticancer activities mediated through the p53-MDM2 interaction is presented in Table 2(Tab. 2) (References in Table 2: Bin et al., 2019[15]; Brockmueller et al., 2023[21]; Chan et al., 2013[28]; Chen and Zhong, 2011[31]; Chen et al., 2022[32][33]; Ding et al., 2012[42]; Fang et al., 2005[48]; Ferraz da Costa et al., 2012[51]; Gao et al., 2020[58]; Giacomelli et al., 2016[62]; Gopal et al., 2009[70]; Gu et al., 2008[72]; Hsieh et al., 2011[83]; Hsu et al., 2009[84]; Huang et al., 2013[87]; Jiang-Jiang et al., 2016[93]; Jin et al., 2013[94]; Kong et al., 2016[104]; Kuo et al., 2002[107]; Li et al., 2005[118], 2007[119], 2015[122]; Liu et al., 2013[124], 2019[126]; Mertens-Talcott et al., 2005[138]; Mu et al., 2009[145]; Nasim and Crooks, 2008[152]; Navarro et al., 2017[153]; Proietti et al., 2014[171]; Qin et al., 2014[174], 2016[173], 2017[172]; Rivera et al., 2017[187]; Rong et al., 2009[189]; She et al., 2001[200]; Sherr, 1998[202]; Song et al., 2018[207]; Srivastava et al., 2007[208]; Subash-Babu et al., 2017[211]; Tanigawa et al., 2008[219]; Tao et al., 2022[220]; Vidya Priyadarsini et al., 2010[230]; Volate et al., 2010[233]; Wang et al., 2008[238], 2008[239], 2012[240], 2014[236]; Wei et al., 2023[243]; Xiong et al., 2017[248]; Yang et al., 2016[254]; Zhai et al., 2008[260]; Zhang et al., 2010[263], 2022[267]; Zheng et al., 2005[273]; Zhu et al., 2019[276]).

## Synergistic and Combinatorial Approaches Using Natural Products

### Rationale for combining different natural products or natural products with conventional cancer therapies

Natural compounds are an inexhaustible source of potential pharmaceuticals (Bhagani et al., 2020[13]; Hossain et al., 2022[79]; Popović-Djordjević et al., 2022[163]; Posadino et al., 2023[167][168], 2024[169]; Ramli et al., 2023[181][182], 2024[180]). In this context, it is becoming increasingly evident that combining different natural products may produce a synergistic effect, which is greater than the sum of their individual effects. This synergy can enhance their health-related properties, such as antioxidant potential and the ability to inhibit cancer cell growth. The combination of various natural compounds also increases the likelihood of simultaneously targeting multiple signaling pathways, thereby improving the chances of inhibiting cancer progression by affecting several stages, including apoptosis, cell proliferation, angiogenesis, and metastasis. 

Synergistic combinations can achieve the desired therapeutic effect at lower doses, thus reducing the risk of side effects and toxicity compared to high doses of a single natural compound, which generally exhibit lower toxicity than synthetic drugs. Furthermore, the interaction between different natural compounds may improve the bioavailability and absorption of each compound, enhancing their overall effectiveness. Combining natural compounds may also reduce the potential for cancer cells to develop resistance, a common issue with single-agent therapies. In addition, natural compounds can be used in conjunction with conventional cancer treatments, such as chemotherapy and radiotherapy, to augment their efficacy.

### Evidence supporting the synergistic effects of natural compounds on the p53-MDM2 pathway and cancer cell proliferation

A study by Li et al. (2021[116]) demonstrated that the administration of green tea polyphenols combined with broccoli sprouts inhibited cancer cell growth by inducing apoptosis and cell cycle arrest in HER2/neu transgenic mice. At the molecular level, the authors highlighted that this combination upregulated the expression of phosphatase and tensin homolog (PTEN), p53, and p16, while downregulating the myelocytomatosis oncogene (MYC), polycomb ring finger oncogene Bmi1, and the reverse transcriptase of telomerase, compared to the control group (Li et al., 2021[121]). A similar study used a transgenic mouse model to investigate the effect of combining withaferin A-rich Ashwagandha and sulforaphane-rich broccoli sprouts on breast cancer prevention (Rahman et al., 2024[178]).

The authors' results revealed that this combination contributes to reducing tumor growth by upregulating the apoptosis-associated proteins (BAX and PUMA), the tumor suppressors (P53, P57), and the BAX:BCL-2 ratio (Rahman et al., 2024[178]). Luo et al. (2020[127]) demonstrated that a mixture of epigallocatechin gallate and doxorubicin upregulated p53 and downregulated MDM2 expression, leading to inhibition of proliferation, induction of doxorubicin-mediated apoptosis, and decreased migration of bladder cancer cells (T24 and SW780) (Luo et al., 2020[127]). Another study revealed the synergistic effect of triptolide and Nutlin-3a (an MDM2 inhibitor) in inhibiting cell proliferation and triggering mitochondrial-mediated apoptosis in vitro and ex vivo in wild-type p53 AML xenograft leukemia cells. This combination delayed tumor growth and reduced the leukemia burden by decreasing mRNA levels of XIAP and Mcl-1 in wild-type p53 cells (Chen et al., 2022[32]).

Icaritin is a naturally occurring flavonoid derived from the Epimedium plant, commonly known as Horny Goat Weed. This plant is rich in several classes of flavonoids, each with specific biological functions (Zhuang et al., 2023[277]). Li and colleagues evaluated the role of icaritin in promoting controlled cell death and inhibiting the proliferation of hepatic cells via the P53/MDM2 and AFP pathways. They discovered that the introduction of icaritin enhanced p53 activity by extending the duration of its response, thereby repressing the expression of AFP genes. Additionally, icaritin stabilized p53, preventing the expression of MDM2 (Li et al., 2021[116]). 

An *in vitro* anticancer analysis using the human glioblastoma cell line model (U87MG) demonstrated that carnosol, a phenolic diterpene found in rosemary, regulated cellular proliferation by increasing intracellular p53 levels, promoting its transcriptional reactivation, degrading the p53-MDM2 interaction, and inducing apoptosis and cell cycle arrest. In the same study, combining carnosol with temozolomide resulted in a synergistic effect, reducing the recurrence of tumor cell proliferation even after the drug was withdrawn (Giacomelli et al., 2016[62]). Table 3(Tab. 3) (References in Table 3: Chen et al., 2022[32]; Giacomelli et al., 2016[62]; Li et al., 2021[121]; Luo et al., 2020[127]; Rahman et al., 2024[177]) summarizes studies demonstrating the synergistic effects of natural products on the p53-MDM2 interaction in various experimental cancer models.

### Strategies for optimizing combination therapies regarding dosage, timing and delivery

While generally considered safe in terms of toxicity, natural compounds can become harmful to cells and the body depending on their concentration and environmental conditions (Pasciu et al., 2010[159]; Giordo et al., 2013[64]; Posadino et al., 2013[164], 2015[165], 2019[166]; Shaito et al., 2020[196]). In this regard, determining the optimal concentrations of these compounds is an important step. This can be achieved by conducting dose-response experiments to identify the concentrations at which each compound is most effective in vitro, as well as preclinical studies to establish the effective dose range for each compound. Identifying combinations that exhibit synergistic effects, which allow for lower doses of each compound while maintaining or enhancing efficacy, is essential. The potential toxicity of mixtures should also be checked by conducting *in vitro* and *in vivo* toxicity studies to ensure that the combined compounds do not exceed toxicity thresholds. Dosages should be adjusted to minimize adverse effects while maintaining therapeutic benefits. Further optimization could involve exploring the timing of compound administration, which may include: i) *Sequential Administration:* Analyzing the effects of compounds administered in a specific sequence. ii) *Simultaneous Administration:* Analyzing the effects of compounds when administered simultaneously to target multiple pathways at once. iii) *Chronotherapy*: Analyzing the effects of compounds when administered in alignment with the body's biological rhythms (circadian rhythms) to enhance efficacy and reduce side effects. Additional optimization may include improving compound delivery. This can involve using nanoparticles, liposomes, or other delivery systems to target compounds specifically to cancer cells, thereby enhancing efficacy and reducing systemic toxicity (Sanna et al., 2011[193]; Quispe et al., 2021[175]; Giordo et al., 2022[67]). Biodegradable controlled-release formulations can also be developed to provide controlled release of compounds over time, maintaining therapeutic levels and reducing the frequency of administration. In this context, the use of combined delivery vehicles may be explored to carry multiple compounds, ensuring they.

## Challenges and Future Directions

Targeting the P53-MDM2 pathway in cancer using natural compounds poses several challenges. The intricate nature of the P53-MDM2 interaction, which encompasses several complex regulatory mechanisms and feedback loops, necessitates a thorough understanding of these interactions to develop effective therapies. Detailed mechanistic studies are necessary to uncover how natural compounds affect the P53-MDM2 pathway and related cellular processes. Variability in the biological activity and therapeutic efficacy of natural products due to differences in plant sources, cultivation conditions, and extraction methods poses a significant challenge, resulting in reproducibility issues (Maaliki et al., 2019[128]; Shaito et al., 2020[196][197]; Alsamri et al., 2021[3]). Moreover, their poor bioavailability and stability hinder many natural compounds' clinical efficacy. Addressing these issues necessitates the creation of advanced delivery systems, including nanoparticles and liposomes, and other nanocarriers, to enhance the delivery and absorption of these compounds (Shaito et al., 2020[196][197]; Posadino et al., 2024[169]).

Although natural products are perceived as safer alternatives to synthetic drugs, they can still cause toxicity and adverse effects at therapeutic doses (Pasciu et al., 2010[159]; Giordo et al., 2013[64]; Posadino et al., 2013[164], 2015[165], 2019[166]; Shaito et al., 2020[197]). Consequently, ensuring safety requires comprehensive toxicity assessments. In addition, cancer cells can develop resistance to therapies targeting the P53-MDM2 pathway, necessitating an in-depth understanding of resistance mechanisms to develop effective counterstrategies. The absence of standardized protocols for the extraction, preparation, and clinical evaluation of natural products further complicates their development and approval as anticancer therapies. Advanced screening methods are essential to overcome many of these challenges. High-throughput screening and computational modeling can aid in identifying and optimizing natural compounds that effectively target the P53-MDM2 pathway. These methods can also facilitate the exploration of synergistic effects when natural compounds are combined with each other or with conventional cancer therapies, potentially enhancing efficacy and reducing side effects. Preclinical and clinical trials are vital to assess the safety, efficacy, and optimal dosage of natural compounds targeting the P53-MDM2 pathway, facilitating the translation of promising compounds from bench to bedside. Personalized medicine approaches, which tailor natural compound-based therapies to individual patients' genetic and molecular profiles, hold promise for improving treatment outcomes. Finally, efforts towards international regulatory harmonization are necessary to streamline the approval process for natural compound-based therapies, thereby facilitating their development and accessibility to patients.

## Conclusion

The intricate interplay between the p53 tumor suppressor and MDM2 ubiquitin ligase is vital for regulating cell cycle, apoptosis, and genomic stability. Disruption of this interaction is a hallmark of various cancers, contributing to poor prognosis and resistance to conventional therapies. An in-depth understanding of this interaction's molecular mechanisms may yield valuable insights into potential therapeutic targets. Natural products have emerged as promising candidates for targeting the p53-MDM2 pathway, offering various compounds capable of modulating this important interaction. Polyphenols, terpenoids, and alkaloids have shown significant potential in inhibiting MDM2 expression, preventing the p53-MDM2 binding, and stabilizing p53, thereby restoring its tumor suppressor functions. These compounds not only exhibit anticancer properties but also lay the groundwork for the development of novel chemotherapeutic agents with reduced toxicity. Despite the promising results, translating these findings into clinical applications remains challenging. These natural compounds' bioavailability, efficacy, and safety need to be addressed through rigorous preclinical and clinical studies. Furthermore, a deeper understanding of the complex regulatory networks involving p53 and MDM2 will aid in the creation of more effective therapeutic strategies. In conclusion, the p53-MDM2 axis represents an important target in cancer therapy, and natural products offer a valuable reservoir of bioactive compounds for therapeutic intervention. Continued research in this area holds great promise for developing innovative treatments that can improve cancer prognosis and patient outcomes.

## Notes

Daniela Calina, Javad Sharifi-Rad (Universidad Espíritu Santo, Samborondón 092301, Ecuador; E-mail: javad.sharifirad@gmail.com) and Gianfranco Pintus (Department of Biomedical Sciences, University of Sassari, Viale San Pietro 43B, 07100 Sassari, Italy; E-mail: gpintus@uniss.it) contributed equally as corresponding author.

## Declaration

### Competing interests

The authors wish to confirm that there are no known conflicts of interest associated with this publication and that there has been no significant financial support for this work that could have influenced its outcome.

### Funding

This work has been developed within the framework of the project eINS-Ecosystem of Innovation for Next Generation Sardinia (cod. ECS 00000038), funded by the Italian Ministry for Research and Education (MUR) under the National Recovery and Resilience Plan (PNRR). This work was also made possible thanks to “Progetto Fondazione di Sardegna -Bando 2022-2023” and “DM 737/2021 resources 2021-2022, funded by the European Union-NextGenerationEU”.

### Acknowledgments

The authors would like to express their gratitude to: Dr. Irina Zamfir, MD, MRCP London, Basildon University Hospital UK for providing professional English editing of this manuscript and for editorial support.

## Figures and Tables

**Table 1:**
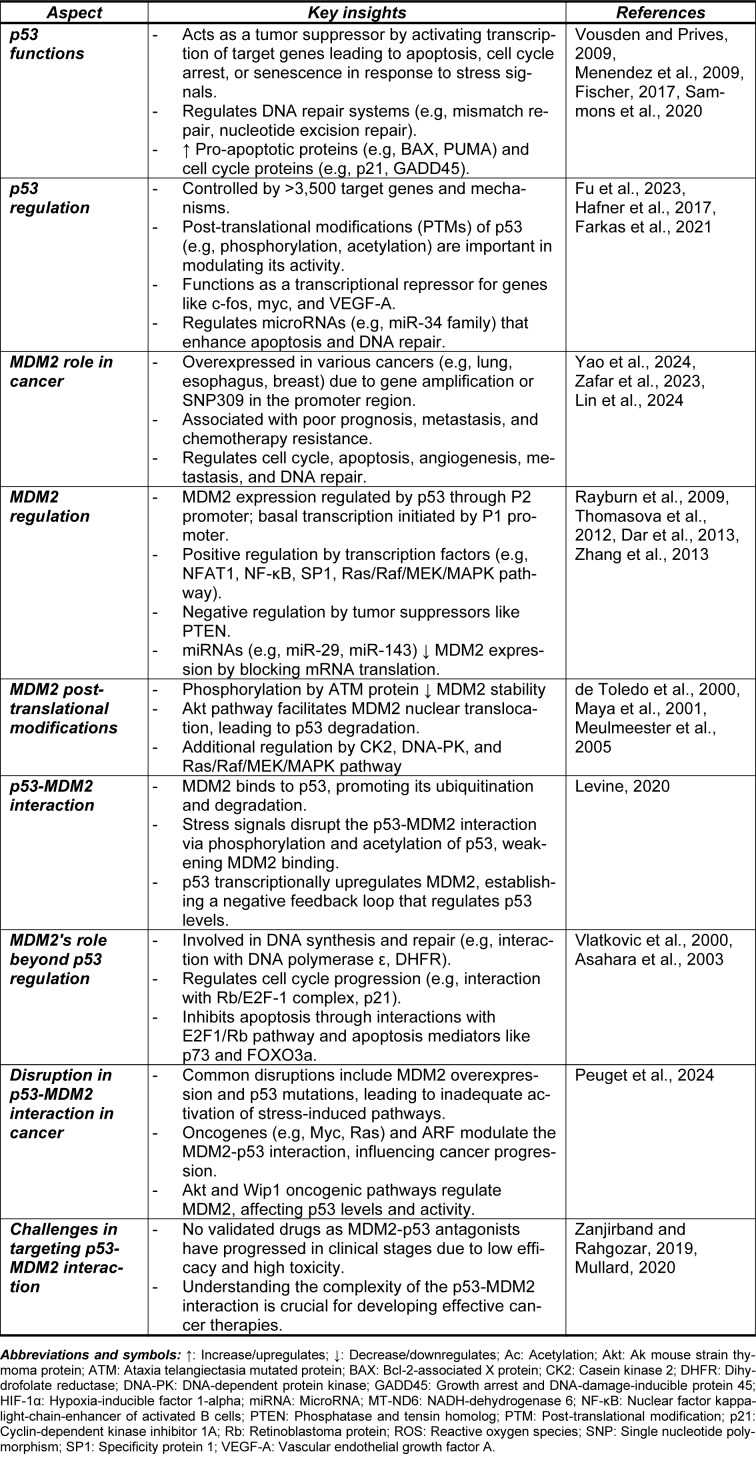
Comprehensive overview of p53-MDM2 biology, regulation, and interaction in cancer

**Table 2:**
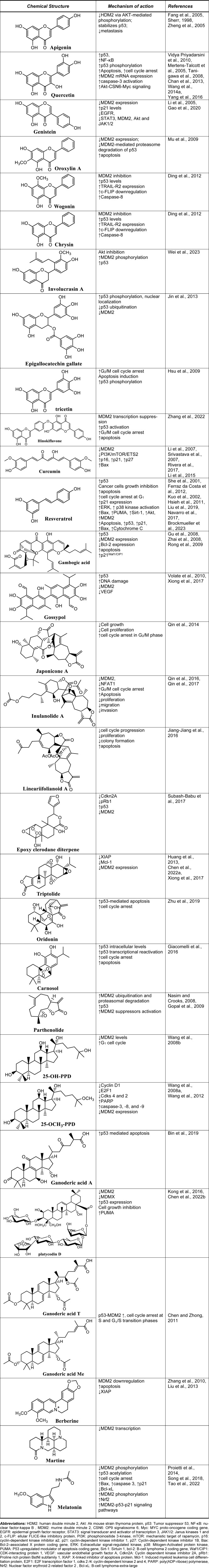
Mechanistic actions and binding modes of some natural compounds that were found to modulate the p53-MDM2 interaction

**Table 3:**
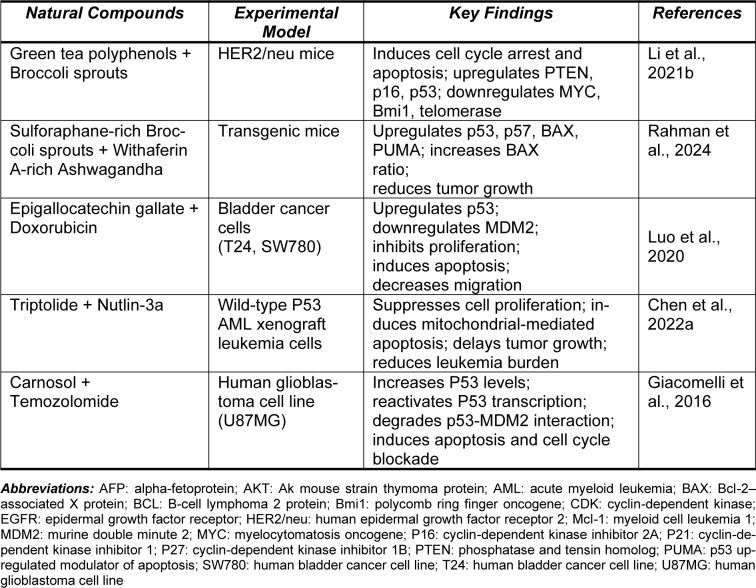
Table 3*:* Synergistic effects of natural compounds on the p53-MDM2 pathway and cancer cell proliferation
